# Selective targeting of JAK/STAT signaling is potentiated by Bcl-xL blockade in IL-2-dependent adult T-cell leukemia

**DOI:** 10.1186/1742-4690-12-S1-O42

**Published:** 2015-08-28

**Authors:** Meili Zhang, Lesley A Mathews Griner, Wei Ju, Damien Y Duveau, Rajarshi Guha, Michael Petrus, Bernard Wen, Michiyuki Maeda, Paul Shinn, Marc Ferrer, Kevin C Conlon, Richard Bamford, John J O'Shea, Craig J Thomas, Thomas A Waldmann

**Affiliations:** 1 Lymphoid Malignancies Branch, Center for Cancer Research, National Cancer Institute, National Institutes of Health, Bethesda, MD, USA; 2 Division of Preclinical Innovation, National Center for Advancing Translational Sciences, National Institutes of Health, Rockville, MD, USA; 3 Laboratory Animal Science Program, Leidos Biomedical Research, Inc., Frederick, Maryland, USA; 4 Kyoto University, Sakyo-ku, Kyoto, Japan; 5 Transponics, Jacobus, PA, USA; 6 Molecular Immunology and Inflammation Branch, National Institute of Arthritis and Musculoskeletal and Skin Diseases, National Institutes of Health, Bethesda, MD, USA

## 

Adult T-cell leukemia (ATL) develops in individuals infected with human T-cell lymphotropic virus-1 (HTLV-1). Presently there is no curative therapy for ATL. The HTLV-1-encoded protein Tax up-regulates Bcl-xL expression and constitutively activates interleukin-2 (IL-2), IL-9, and IL-15 autocrine/paracrine systems resulting in amplified JAK/STAT signaling. Consequently, inhibition of JAK signaling reduces ex vivo proliferation of PBMCs from ATL patients in smoldering and chronic stages. Currently, two JAK inhibitors are approved for human use. In this study, we examined activity of multiple JAK inhibitors in IL-2-dependent and IL-2-independent ATL cell lines. The highly selective JAK inhibitor ruxolitinib was examined in a high-throughput matrix screen and the Bcl-2/Bcl-xL inhibitor navitoclax was identified as a strong candidate for multicomponent therapy. An examination of the mechanistic underpinnings of this combination highlighted a stimulation of Bim and PUMA expression and reduced phosphorylation of BAD upon cellular exposure to ruxolitinib. The combination was noted to strongly activate BAX, effect mitochondrial depolarization and increase caspase 3/7 activity that leads to PARP and Mcl-1 cleavage. Ruxolitinib and navitoclax independently demonstrated modest antitumor efficacy while the combination dramatically lowered tumor burden and prolonged survival in an aggressive ATL murine model. Critically, this combination strongly blocked ex vivo proliferation of five ATL patients’ PBMCs. These studies provide support for a therapeutic trial in patients with smoldering and chronic ATL using a drug combination that inhibits JAK signaling and anti-apoptotic protein Bcl-xL.

